# Health seeking behaviour and self-care among hypertensive and diabetics at risk of CKD in Buea, Cameroon

**DOI:** 10.4102/jphia.v16i1.1427

**Published:** 2025-12-09

**Authors:** Sally E. Mondoa, Emmanuel Y. Vubo, Nsagha D. Shey

**Affiliations:** 1 Department of Public Health and Hygiene, Faculty of Health Sciences, University of Buea, Buea, Cameroon; 2 Department of Sociology, Faculty of Social and Management Sciences, University of Buea, Buea, Cameroon

**Keywords:** chronic kidney disease, CKD, hypertension and diabetes, self-care practice, healthcare-seeking behaviour

## Abstract

**Background:**

Chronic kidney disease (CKD) has emerged as a major public health concern in Cameroon, particularly among individuals with hypertension and diabetes, who face significant barriers to effective disease management.

**Aim:**

This prospective cohort study aimed to assess CKD awareness, healthcare-seeking behaviours, and self-care management practices among hypertensive and/or diabetic patients at risk of CKD.

**Setting:**

The study was conducted from September 2022 to April 2024 among 400 participants attending four healthcare facilities in the Buea Health District, Cameroon.

**Methods:**

Structured questionnaires collected data on socio-demographics, clinical status, CKD knowledge and self-care adherence, with logistic regression analysing associations between risk factors and outcomes.

**Results:**

The study revealed alarming gaps in CKD knowledge, with only 35% of participants recognising hypertension and diabetes as primary risk factors. Misconceptions were widespread, including 90% falsely believing urine colour reliably indicates kidney health. Self-care practices were inconsistent: while dietary adherence was relatively high (97.3% avoided excess salt, 88.5% consumed vegetables regularly), critical monitoring behaviours were neglected – only 15.2% underwent regular renal check-ups, and 29.8% monitored blood pressure at home. Hypertension was strongly associated with CKD (adjusted odds ratio [OR] = 4.28, 95% confidence interval [CI]: 1.25–14.67), whereas diabetes alone showed no significant link. Socio-demographic disparities further compounded these challenges, with tertiary-educated participants demonstrating better CKD awareness than those with primary education (*p* < 0.05).

**Conclusion:**

These findings underscore systemic deficiencies in CKD prevention and management, including poor health literacy, financial barriers and inadequate healthcare infrastructure.

**Contribution:**

To mitigate CKD’s growing burden, policymakers must prioritise community-based education, subsidised screening programmes and improved access to monitoring tools. Culturally tailored interventions, integrating patient empowerment and health system strengthening, are urgently needed to enhance early detection and long-term outcomes in resource-limited settings like Cameroon.

## Introduction

Chronic kidney disease (CKD) has emerged as a critical global health challenge, with prevalence rates increasing dramatically in recent decades. The Global Burden of Disease Study estimates that over 850 million people worldwide suffer from kidney diseases, including approximately 700 million with diagnosed CKD.^1,2^ This escalating burden presents particular challenges in low- and middle-income countries (LMICs), where healthcare systems often lack adequate resources for effective CKD management.^3,4^ The disease’s strong association with cardiovascular complications and other non-communicable diseases (NCDs) places additional strain on already overburdened health systems.^5,6^

The epidemiological profile of CKD reveals its devastating impact on population health. Hypertension and diabetes mellitus (DM) – both experiencing global increases – account for the majority of CKD cases worldwide.^7,8^ The condition’s progression leads to significantly reduced quality of life, increased mortality rates and substantial healthcare expenditures.^9^ Nowhere are these consequences more severe than in sub-Saharan Africa, where limited access to diagnostic services and treatment options creates particularly dire outcomes.^10,11^

Cameroon exemplifies these regional challenges, with CKD emerging as a leading cause of hospital admissions and outpatient visits.^12^ Recent epidemiological studies estimate that 10% – 15% of Cameroon’s adult population lives with CKD, with hypertension (40% – 60% of cases) and diabetes (20% – 30%) identified as predominant comorbidities.^7,11^ Alarmingly, research has primarily focused on diagnosed patients, leaving a critical knowledge gap regarding at-risk populations.^13^ This oversight is particularly concerning given the low levels of CKD awareness (30% – 40%) among high-risk groups and the substantial barriers to early detection.^14^

The management of CKD in Cameroon faces multiple systemic challenges. For advanced cases requiring renal replacement therapy, adherence to haemodialysis remains distressingly low because of financial constraints and limited treatment availability.^12,15^ These realities underscore the urgent need for effective preventive strategies and early intervention programmes.^16^ Healthcare-seeking behaviours and self-care practices among individuals with hypertension and diabetes – the primary CKD risk factors – therefore assume critical importance.^17,18^

Theoretical frameworks offer valuable insights for addressing these challenges. Orem’s self-care deficit nursing theory emphasises the crucial role of patient capability in performing self-care activities, with healthcare providers supplementing when deficiencies exist.^19^ Complementing this, Self-Determination Theory highlights how fostering autonomy, competence and relatedness can enhance motivation for sustained self-care behaviours.^20^ These models collectively suggest that patient education, empowerment and participatory decision-making constitute essential components of effective CKD prevention.^21^

However, the Cameroonian context presents unique obstacles to optimal self-care implementation. Multifaceted barriers, including poverty, healthcare access limitations, inadequate health literacy and cultural beliefs, significantly hinder prevention efforts.^16,22^ Recent studies in the Buea region reveal particularly concerning findings: only 25% of diabetic patients adhere to recommended self-care protocols, with even fewer regularly monitoring kidney function.^13,21^ These deficiencies highlight the urgent need for context-specific interventions addressing both clinical and socioeconomic determinants of CKD outcomes.

This study aims to bridge critical knowledge gaps by examining healthcare-seeking behaviours and self-care management practices among at-risk hypertensive and diabetic populations in Cameroon’s Buea Health District. Specifically, the investigation identified community-dwelling individuals with hypertension and diabetes, assessed their knowledge of CKD and awareness of risk factors and characterised existing self-care practices. The findings will inform targeted, culturally appropriate interventions to enhance CKD prevention and management in this vulnerable population through integrated approaches encompassing patient education, health system strengthening and policy reforms.^16,23^

## Materials and methods

### Study design

This was a prospective cohort study that followed participants over time to assess how baseline exposures influence the development of health outcomes. The study enrolled 400 hypertensive and/or diabetic patients from four healthcare facilities in Buea, Cameroon (September 2022 – April 2024). Using interviewer-administered questionnaires, we collected data on demographic characteristics, clinical status and knowledge of CKD risk factors. Participants received standardised health education on preventive self-care practices using World Health Organization (WHO) guidelines during clinic visits.

### Study setting

**FIGURE 1:**
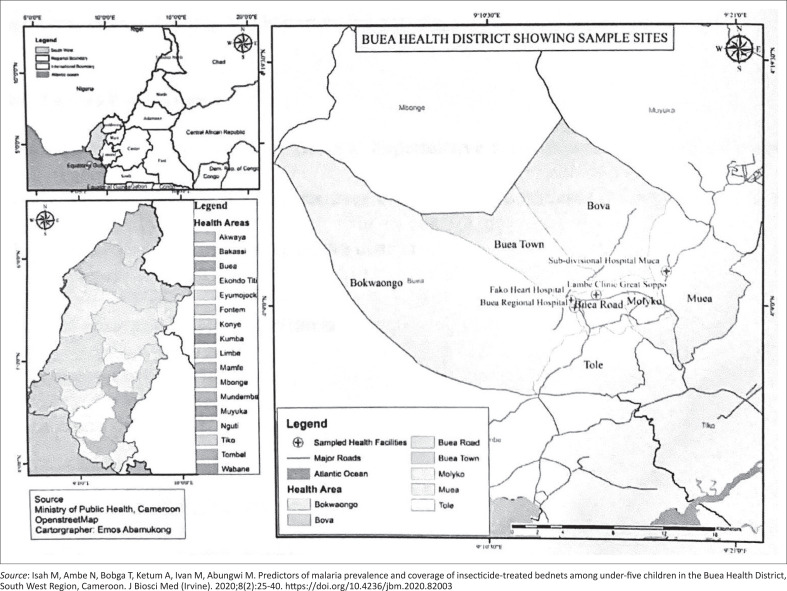
Map of Buea Health District.

The study was conducted at four facilities in Buea Health District, Southwest Region, Cameroon: Buea Regional Hospital, Muea Sub-Divisional Hospital, Fako Heart Hospital and Lambe Clinic (Great Soppo) (Figure 1).^24^

Buea is situated on the eastern slopes of Mount Cameroon (4040 m), the highest peak in West and Central Africa. The region experiences distinct wet (between March and October) and dry (between November and February) seasons.

### Study population and sample size

The sample size was determined using standard statistical methods for estimating a single population proportion. A 95% confidence level, a margin of error of 5% and a population proportion of 0.5 (to account for maximum variability) were used, resulting in a minimum required sample size of 385 participants. This yielded 384 participants, which we increased to 400 (accounting for 5% attrition). Facility-specific sample sizes were determined through probability proportionate to size allocation.

### Data collection

Participants were consecutively recruited from routine diabetes and/or hypertension clinics at the four facilities. Eligible participants were adults aged 18 years and older who had a confirmed diagnosis of hypertension (defined as systolic blood pressure ≥ 140 mmHg or diastolic blood pressure ≥ 90 mmHg, or current use of antihypertensive medication) and/or type 2 diabetes (defined as fasting blood glucose ≥ 126 mg/dL or current use of glucose-lowering medication). Exclusion criteria included advanced CKD (estimated glomerular filtration rate [eGFR] < 30 mL/min/1.73 m^2^) and pregnancy. All participants provided written informed consent prior to enrolment.

Trained research nurses administered pre-tested, structured questionnaires during routine clinic visits. Instruments were available in both English and Pidgin (the local lingua franca) and assessed: Socio-demographic characteristics, self-care practices (medication adherence, alcohol use, physical activity), healthcare-seeking behaviours and exposure to self-care education. Questionnaires were validated through pilot testing at Limbe Regional Hospital (outside the study area).

### Data analysis

Data were cleaned, coded and analysed descriptively (frequencies for categorical variables; measures of central tendency for continuous variables). Binary logistic regression assessed associations between five self-care outcomes (diet, exercise, weight management, medication adherence and behaviours) and three conditions (diabetes, hypertension or both), adjusting for age, sex and education. Adjusted odds ratios (AORs) with 95% confidence intervals (CIs) were calculated (*p* < 0.05 significance). Multivariable analysis controlled for confounders. MINITAB 20.1 was used for all analyses, including model fitting and interaction testing.

### Ethical considerations

Ethical clearance to conduct this study was obtained from the University of Buea (No. 2023/2080-04/UB/SG/IRB/FHS) and administrative clearance from the Southwest Regional Delegation of Public Health (No. p42/MPH/SWR/RDPH/CB.PT/681/519). The study also received facility-level approvals from all participating sites. All participants provided written informed consent prior to enrolment.

## Results

### Socio-demographic characteristics of the participants

The study population comprised 400 participants with diabetes and/or hypertension from four healthcare facilities in Buea Health District, with a mean age of 60.3 years (standard deviation [s.d.] ±11.8). Females represented the majority of participants (70.8%), while males accounted for 29.2% (see Table 1). Age distribution showed that 35.3% of participants were between 61 years and 70 years old, followed by 27.3% in the 51–60 years-old age group and 15.3% aged 71 years – 80 years. Younger age groups were less represented, with only 0.8% aged 20 years – 30 years. Regarding marital status, 56.8% of participants were married, 24.0% were widowed, 17.8% were single and 1.5% were divorced or separated. Educational attainment revealed that 41.3% had primary-level education, 33.3% secondary education and 18.5% tertiary education, while 7.0% had no formal education. Occupation distribution showed 34.8% employed in the private sector, 29.5% unemployed, 21.0% farmers, 13.8% civil servants and 1.0% students. Geographically, 89.0% of participants resided in urban areas compared to 11.0% in rural communities. Financially, the majority (59.5%) earned less than 50 000 FCFA (*Francs Communaute Francaise Africaine* [African Financial Community {Franc}]) monthly, while only 7.0% earned above 200 000 FCFA. In terms of healthcare utilisation, over half of participants (52.3%) received treatment at Buea Regional Hospital, followed by 37.5% at Muea Sub-Divisional Hospital, with smaller proportions attending Fako Heart Centre (5.3%) and Lambe Clinic (5.0%) (see Table 1). This demographic profile highlights several important characteristics of the study population, including the predominance of middle-aged and elderly individuals, females, urban dwellers and those with limited education and income, which may influence healthcare-seeking behaviours and disease management patterns.

**TABLE 1:** Socio-demographic characteristics of study participants in Buea Health District.

Characteristic	Categories	*n*	%
Sex	Female	283	70.8
	Male	117	29.2
Age group (years)	20–30	3	0.8
	31–40	28	7.0
	41–50	47	11.8
	51–60	109	27.3
	61–70	141	35.3
	71–80	61	15.3
	> 80	11	2.8
Marital status	Married	227	56.8
	Single	71	17.8
	Widowed	96	24.0
	Divorced or separated	6	1.5
Education level	None	28	7.0
	Primary	165	41.3
	Secondary	133	33.3
	Tertiary	74	18.5
Occupation	Private sector	139	34.8
	Unemployed	118	29.5
	Farmer	84	21.0
	Civil servant	55	13.8
	Student	4	1.0
Treatment centre	Buea Regional Hospital	209	52.3
	Muea Sub-Divisional	150	37.5
	Fako Heart Centre	21	5.3
	Lambe Clinic	20	5.0
Residence	Urban	356	89.0
	Rural	44	11.0
Monthly salary (FCFA)	< 50 000	238	59.5
	50 000–99 000	71	17.7
	100 000–149 000	40	10.0
	150 000–199 000	23	5.8
	> 200 000	28	7.0

FCFA, *French Communaute Francaise Africaine (African Financial Community [Franc])*.

### Prevalence of diabetes, hypertension, comorbidity and the associated clinical signs and symptoms

The study revealed a 26% prevalence of DM and a 33% prevalence of hypertension (HTN) among participants, with 41% having both conditions. Most diabetic patients (52.4%) were diagnosed ≥ 4 years prior, while 62% of hypertensive participants had HTN for ≥ 4 years. Clinical measurements showed 59% had high blood pressure and 77.75% had elevated fasting blood sugar levels (see Table 2). The data demonstrate significant disease burden, with nearly three-quarters of participants presenting abnormal glycaemic control and over half showing uncontrolled hypertension, particularly among those with longer disease duration.

**TABLE 2:** Prevalence and clinical characteristics of diabetes, hypertension and comorbidity in Buea Health District (*N* = 400).

Clinical parameter	Category	*n*	%
**Diabetes mellitus (DM)**			
DM diagnosis	No	296	74.0
	Yes	104	26.0
Duration of DM diagnosis	0–1 year	98	24.5
	2–3 years	92	23.0
	≥ 4 years	210	52.5
**Hypertension (HTN)**			
HTN diagnosis	No	268	67.0
	Yes	132	33.0
**Comorbidity**			
HTN and DM coexistence	No	232	58.0
	Yes	164	41.0
**Clinical measurements**			
Blood pressure status	High	236	59.0
	Normal	164	41.0
Fasting blood sugar	High	311	77.8
	Normal	90	22.5
HTN diagnosis duration	0–1 year	58	14.5
	2–3 years	94	23.5
	≥ 4 years	248	62.0

### Participant knowledge of chronic kidney disease risk factors in Buea Health District

Knowledge assessment revealed significant gaps in CKD awareness among participants. Only 35% recognised diabetes and hypertension as CKD risk factors, while 63% correctly identified long-term alcohol consumption as harmful. Most participants (73%) understood early detection reduces healthcare costs, but misconceptions persisted: 90% erroneously believed kidney problems could be detected through urine colour and/or smell, and 94% didn’t associate obesity with CKD risk. Notably, 98% correctly rejected water consumption as a cause, yet 72% were unaware CKD can be asymptomatic in advanced stages (see Table 3). These findings highlight critical knowledge deficits regarding both risk factors (16% recognised smoking risks) and disease presentation (only 28% knew about asymptomatic progression) (see Table 3).

**TABLE 3:** Knowledge of chronic kidney disease risk factors among hypertensive and diabetic patients in Buea Health District (*N* = 400).

Knowledge statement	Yes		No	
	*n*	%	*n*	%
**Risk factor identification**				
DM/HBP can cause CKD	141	35.3	259	64.7
Smoking increases CKD risk	64	16.0	336	84.0
Obesity leads to CKD	24	6.0	376	94.0
Long-term alcohol causes CKD	251	62.8	149	37.2
**Disease manifestation**				
CKD can be asymptomatic	111	27.8	289	72.2
Polyuria indicates CKD	19	4.8	-	-
Anaemia and cardiovascular disorder are risky for CKD	108	27.0	292	73.0
**Misconceptions**				
Water intake causes CKD	-	-	393	98.2
Urine colour detects CKD	40	10.0	360	90.0
**Prevention knowledge**				
Early detection saves costs	293	73.2	107	26.8

DM, diabetes mellitus; HBP, high blood pressure; CKD, chronic kidney disease.

### Socio-demographic characteristics in association with knowledge of risk factors of chronic kidney disease among participants

The study revealed significant associations between socio-demographic factors and CKD knowledge. Older adults (41 years – 60 years) demonstrated better awareness that long-term alcohol use causes CKD (χ^2^ = 15.67, *p* = 0.016), with 62.5% overall recognising this risk (see Table 4). Females showed marginally higher knowledge than males (71.3% vs. 28.7%, χ^2^ = 399.23, *p* < 0.001) regarding urine-based detection misconceptions. Facility-based differences emerged, with only 1.5% at Fako Heart Centre recognising DM/HTN as CKD risks versus 16.8% at Regional Hospital (χ^2^ = 435.26, *p* < 0.001). Married participants exhibited greater alcohol-CKD awareness (38.3%) than widowed individuals (11%) (see Table 4). Education significantly impacted understanding, as 94.5% with primary/secondary education believed untreated malaria causes CKD versus 5.5% with tertiary education (χ^2^ = 407.31, *p* < 0.001) (see Table 4). Occupation also influenced perceptions, with 90% of private sector workers endorsing urine colour as a detection method compared to 10% of unemployed participants (χ^2^ = 424.21, *p* < 0.001) (see Table 4). These findings highlight critical knowledge gaps across demographic groups regarding modifiable CKD risk factors.

**TABLE 4:** Association between socio-demographic factors and chronic kidney disease knowledge among hypertensive and diabetic patients.

Variable	Knowledge item	Sub-variable	No		Yes		Total		χ^2^	*p*-value
			*n*	%	*n*	%	*n*	%		
Age (years)	Long-term alcohol causes CKD	-	-	-	-	-	-	-	15.667	0.016
		20–30	6	1.5	22	5.5	28	7.0	-	-
		31–40	16	4.0	32	8.0	48	12.0	-	-
		41–50	32	8.0	77	19.3	109	27.3	-	-
		51–60	58	14.5	79	19.8	137	34.3	-	-
		61–70	31	7.8	30	7.5	61	15.3	-	-
		71–80	6	1.5	8	2.0	14	3.5	-	-
Sex	Urine colour detects kidney problems	-	-	-	-	-	-	-	399.234	< 0.001
		Female	104	26.0	179	44.8	283	70.8	-	-
		Male	45	11.3	72	18.0	117	29.3	-	-
Facility	DM/HTN causes CKD	-	-	-	-	-	-	-	435.257	< 0.001
		Fako Heart Centre	67	16.8	8	2.0	75	18.8	-	-
		Lambe Clinic	68	17.0	38	9.5	106	26.5	-	-
		Regional Hospital	68	17.0	68	17.0	136	34.0	-	-
		Muea Sub-Divisional	56	14.0	27	6.8	83	20.8	-	-
Marital Status	Long-term alcohol causes CKD	-	-	-	-	-	-	-	414.680	< 0.001
		Married	73	18.3	154	38.5	227	56.8	-	-
		Single	23	5.8	46	11.5	69	17.3	-	-
		Widowed	52	13.0	44	11.0	96	24.0	-	-
		Divorced or separated	1	0.3	7	1.8	8	2.0	-	-
Education	Untreated malaria causes CKD	-	-	-	-	-	-	-	407.305	< 0.001
		No formal education	30	7.5	0	0.0	30	7.5	-	-
		Primary	158	39.5	8	2.0	166	41.5	-	-
		Secondary	124	31.0	7	1.8	131	32.8	-	-
		Tertiary	66	16.5	7	1.8	73	18.3	-	-
Occupation	Urine colour detects kidney problems	-	-	-	-	-	-	-	424.209	< 0.001
		Private sector	125	31.3	14	3.5	139	34.8	-	-
		Unemployed	115	28.8	3	0.8	118	29.5	-	-
		Farmer	77	19.3	7	1.8	84	21.0	-	-
		Civil servant	41	10.3	14	3.5	55	13.8	-	-
		Student	2	0.5	2	0.5	-	-	-	-

CKD, chronic kidney disease; HTN, hypertension; DM, diabetes mellitus.

### Self-care management practices among study participants

The study revealed mixed self-care practices among participants (see Table 5). While dietary practices showed relatively good adherence – 97.3% avoided high-salt foods and 88.5% consumed vegetables regularly – concerning gaps existed in other areas. Only 56.5% engaged in regular exercise, with 41.8% never exercising. Medical adherence was suboptimal, with 20.5% forgetting medications and 25% missing appointments. Critical monitoring practices were particularly neglected: 84.8% lacked regular renal check-ups, and 70.2% didn’t monitor blood pressure at home. Paradoxically, 92.5% reported adding salt to food despite 97.3% claiming to avoid salty foods. Exercise frequency varied significantly, with just 10.5% exercising daily. These findings highlight a disconnect between knowledge and practice, particularly regarding cardiovascular risk monitoring and consistent lifestyle modifications. The low rates of renal monitoring (15.2%) and home BP checks (29.8%) are especially concerning given participants’ high-risk status for CKD progression (see Table 5).

**TABLE 5:** Self-care management practices among hypertensive and diabetic patients in Buea Health District.

Variable	Practice	Adherent		Non-adherent	
		*n*	%	*n*	%
Category	Activity	226	56.5	174	43.5
Dietary practices	Low salt intake	389	97.3	11	2.8
	Vegetable consumption	354	88.5	46	11.5
	Avoid processed foods	229	57.2	171	42.8
	No added salt	30	7.5	370	92.5
Medical adherence	Medication compliance	318	79.5	82	20.5
	Appointment keeping	300	75.0	100	25.0
	Home BP monitoring	119	29.8	281	70.2
	Regular renal check-ups	61	15.2	339	84.8

Note: Specific behaviours (where applicable): Never: 167 (41.8%); weekly: 112 (28.0%); 2–3×/week: 79 (19.8%); daily: 42 (10.5%).

BP, blood pressure.

### Self-care management practices and associated risk factors in diabetic and hypertensive patients in Buea Health District

The analysis revealed significant variation in self-care adherence among diabetic-hypertensive patients (see Table 6). Home glucose monitoring showed the strongest association (χ^2^ = 8.64, *p* = 0.003), with 85.2% adherence among regular users versus 56.3% overall. Medication adherence approached significance (*p* = 0.05), with 65.9% adherence among those who occasionally forgot medications versus 56.3% among consistent users. Most practices showed no significant associations: physical activity (*p* = 0.86), renal check-ups (*p* = 0.17) and dietary salt avoidance (*p* = 0.17). Notably, home BP monitoring (59.9% adherent) and vegetable consumption (62.7% adherent) showed moderate but non-significant adherence patterns (see Table 6). The data suggest that while patients generally maintain basic self-care, critical monitoring behaviours like glucose checks vary significantly, potentially reflecting access barriers or knowledge gaps. These findings highlight the need for targeted interventions to improve consistent monitoring practices in this high-risk population.

**TABLE 6:** Association between self-care practices and clinical factors among diabetic-hypertensive patients in Buea Health District.

Self-care practice	Non-adherent		Adherent		Total		χ^2^	*p*-value
	*n*	%	*n*	%	*n*	%		
**Physical activity**								
Regular exercise	73	41.2	104	58.8	177	100	0.034	0.855
Irregular exercise	94	42.2	129	57.8	223	100	-	-
**Medical monitoring**								
Renal check-ups	138	40.4	204	59.6	342	100	1.899	0.168
Home BP monitoring	113	40.1	168	59.9	281	100	3.052	0.384
Home glucose monitoring	163	43.7	210	56.3	373	100	8.638	0.003
**Medication adherence**								
Never miss medications	139	43.7	179	56.3	318	100	3.848	0.050
Sometimes forget medications	28	34.1	54	65.9	82	100	-	-
**Dietary practices**								
Avoid high-salt foods	164	42.4	223	57.6	387	100	1.927	0.165
Regular vegetable intake	19	37.3	32	62.7	51	100	0.486	-

BP, blood pressure.

### Clinical and socio-demographic characteristics in association with chronic kidney diseases

The analysis revealed hypertension as a significant independent risk factor for CKD, with hypertensive patients showing 4.7 times higher crude odds (95% CI: 1.40–16.00) and 4.3 times higher adjusted odds (95% CI: 1.25–14.67) of developing CKD compared to normotensives (see Table 7). Diabetes mellitus alone showed no significant association (crude odds ratio [OR] = 0.39, 95% CI: 0.05–3.06; adjusted OR = 0.32, 95% CI: 0.04–2.74). The comorbidity of diabetes and hypertension demonstrated a non-significant trend towards increased CKD risk (adjusted OR = 1.86, 95% CI: 0.60–5.74) (see Table 7). These findings suggest that hypertension drives CKD risk in this population, while diabetes may require longer duration or poorer control to manifest significant renal effects. The persistent association after adjustment indicates hypertension’s robust relationship with CKD, independent of other measured factors.

**TABLE 7:** Association between metabolic conditions and chronic kidney disease.

Risk factor	Category	Total		Unadjusted analysis		Adjusted analysis	
		*n*	%	OR	95% CI	OR	95% CI
Diabetes mellitus	No (Ref)	64	16.0	1.00	-	1.00	-
	Yes	336	84.0	0.39	0.05–3.06	0.32	0.04–2.74
Hypertension	No (Ref)	35	8.8	1.00	-	1.00	-
	Yes	365	91.3	4.73*	1.40–16.00	4.28	1.25–14.67
HTN+DM comorbidity	No (Ref)	167	41.8	1.00	-	1.00	-
	Yes	233	58.3	1.41	0.48–4.09	1.86	-

HTN, hypertension; DM, diabetes mellitus; OR, odds ratio; CI, confidence interval.

Table 8 presents AORs, CIs and *p*-values for various factors associated with diabetes, hypertension and the coexistence of both conditions. Self-care management such as regular physical exercise were significantly associated with a reduced risk of hypertension (AOR: 0.07, 95% CI: 0.01 to 0.12, *p* = 0.015). However, this variable did not show a significant association with diabetes or with the coexistence of diabetes and hypertension, as indicated by higher *p*-values of 0.506 and 0.731, respectively. Other self-care behaviours, such as regular renal check-ups, salt intake, vegetable consumption and medication adherence, did not demonstrate statistically significant associations with any of the three health outcomes, as all *p*-values exceeded the conventional threshold of 0.05.

**TABLE 8:** Binary logistic regression analysis for the associations between three conditions and self-care management and demographic data of the respondents.

Variables	Diabetes			Hypertension			Diabetes and hypertension		
	AOR	95% CI	*p*-value	AOR	95% CI	*p*-value	AOR	95% CI	*p*-value
**Self-care management**									
Regular physical exercise	0.03	−0.05, 0.11	0.506	0.07	0.01, 0.12	0.015	0.02	−0.09, 0.12	0.731
Regular renal check-up	−0.09	−0.20, 0.02	0.123	−0.00	−0.08, 0.07	0.983	−0.12	−0.26, 0.03	0.126
Eat foods with too much salt	0.09	−0.12, 0.30	0.403	−0.02	−0.17, 0.12	0.767	0.19	−0.10, 0.47	0.199
Regularly eating of vegetables	0.02	−0.09, 0.14	0.702	−0.00	−0.08, 0.08	0.972	−0.03	−0.18, 0.13	0.735
Forget to take my medication	0.037	−0.06, 0.13	0.430	−0.05	−0.02, 0.11	0.170	0.06	−0.07, 0.19	0.342
**Age (years)**									
18–23	0.14	−0.32, 0.61	0.544	−0.14	−0.46, 0.18	0.390	0.43	−0.20, 1.06	0.177
18–28	0.11	−0.54, 0.75	0.747	−0.02	−0.46, 0.43	0.945	−0.57	−1.43, 0.29	0.196
29–39	0.13	−0.05, 0.31	0.160	0.08	−0.05, 0.20	0.230	0.19	−0.05, 0.44	0.124
40–50	−0.01	−0.15, 0.14	0.962	−0.01	−0.11, 0.09	0.835	0.03	−0.17, 0.23	0.744
51–61	−0.05	−0.18, 0.08	0.466	−0.02	−0.11, 0.08	0.714	−0.01	−0.19, 0.17	0.942
62–72	−0.10	−0.23, 0.03	0.141	0.02	−0.07, 0.11	0.678	−0.03	−0.21, 0.15	0.769
73–83	−0.17	−0.32, -0.01	0.035	−0.04	−0.15, 0.07	0.458	−0.11	−0.32, 0.10	0.305
**Gender**									
Male	Ref	-	Ref	Ref	-	Ref	Ref	-	Ref
Female	0.01	−0.03, 0.05	0.601	0.01	−0.02, 0.04	0.620	0.03	−0.02, 0.09	0.228
**Education**									
Formal education	0.07	−0.04, 0.18	0.203	0.02	−0.06, 0.09	0.708	0.01	−0.14, 0.16	0.876
Primary	0.018	−0.04, 0.08	0.577	−0.01	−0.05, 0.03	0.583	0.03	−0.06, 0.11	0.531
Secondary	−0.07	−0.13, -0.01	0.036	0.01	−0.04, 0.05	0.696	−0.02	−0.10, 0.07	0.742

AOR, adjusted odds ratio; CI, confidence interval.

When investigative age groups were considered, most categories did not show significant associations with diabetes, hypertension or both. An exception was observed in the 73–83 years-old age group, where there was a statistically significant negative association with diabetes (AOR: −0.17, 95% CI: −0.32 to −0.01, *p* = 0.035). No significant associations were observed for this age group with hypertension or with the combined outcome (see Table 8).

For gender and education, neither female gender nor levels of education (formal, primary or secondary) were significantly associated with the risk of diabetes, hypertension or both, except for secondary education, which showed a modest but significant negative association with diabetes (AOR: −0.07, 95% CI: −0.13 to −0.01, *p* = 0.036).

### Interaction plot

**FIGURE 2:**
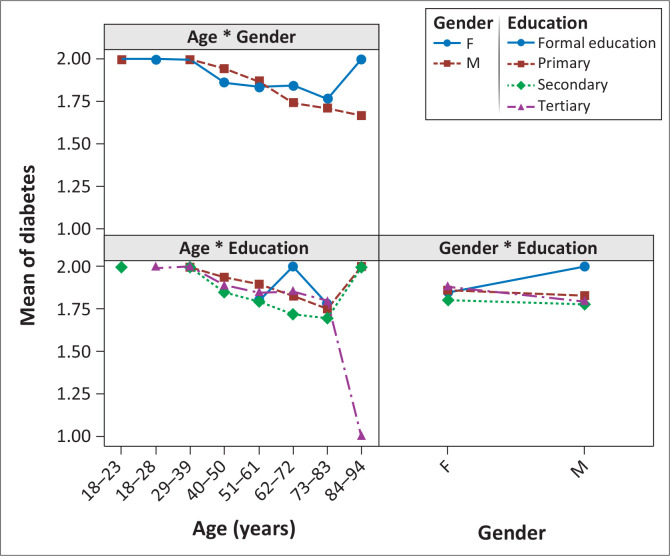
Interaction plot for diabetes against age, gender and education.

The interaction plot (Figure 2) reveals significant trends in diabetes prevalence across age, gender and education levels. The age*gender graph reveals that the mean diabetes levels for females start at around 2.00 in the 18–29 years-old age group and gradually decrease to approximately 1.50 by the 64–94 years-old age range. In contrast, males exhibit a lower starting mean of about 1.75, which also declines to about 1.25 in older age brackets. The gender*education interaction graph indicates that individuals with tertiary education consistently show the lowest diabetes levels, with mean values near 1.50, while those with primary education have higher levels, particularly around 2.00 for younger age groups.

**FIGURE 3:**
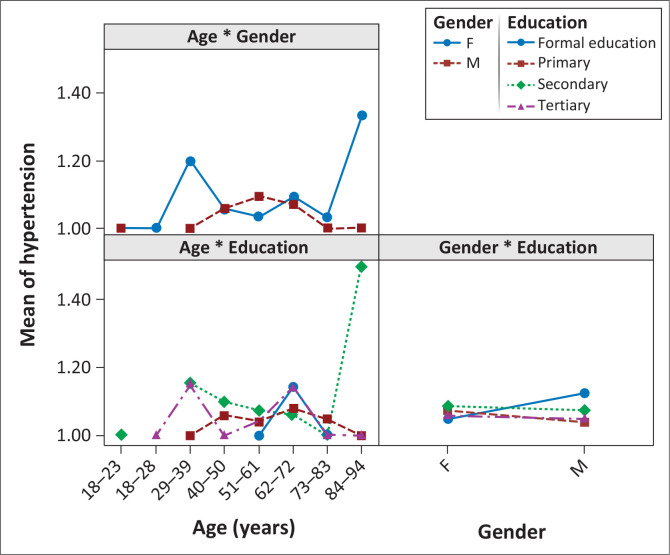
Interaction plot for hypertension against age, gender and education.

The interaction plot for hypertension illustrates distinct trends across age, gender and education levels (Figure 3). The interaction plot of age*gender reveals that females display a peak mean hypertension level of approximately 1.40 in the 40–50 years-old age group, while males show a more gradual increase, reaching a mean of about 1.20 in the same age range before declining slightly (Figure 3). The gender*education interaction highlights educational impacts, where individuals with tertiary education demonstrate the lowest hypertension levels, particularly among females, with a mean near 0.80, while those with primary education show higher levels, particularly in the 29–39 years-old age group, where mean hypertension is around 1.20 (Figure 3).

**FIGURE 4:**
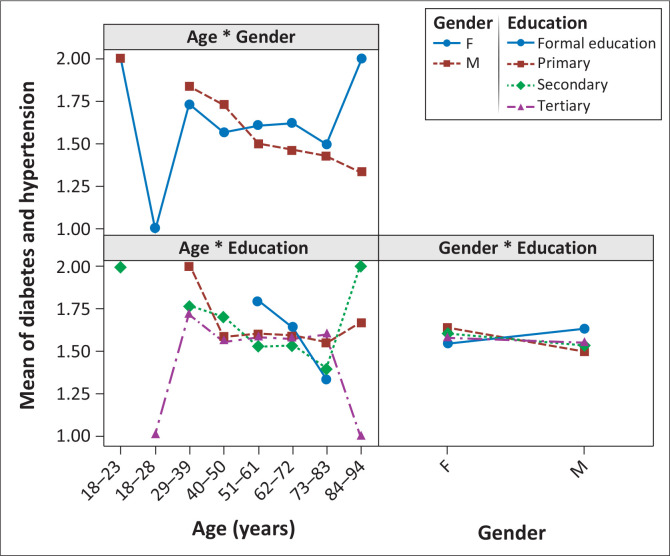
Interaction plot for diabetes and hypertension against age, gender and education.

The interaction plot for diabetes and hypertension showcases the interplay between age, gender and education levels. The plot of age*gender reveals that females have a mean diabetes and hypertension level starting at approximately 2.00 in the 18–29 years-old age group, which decreases to around 1.60 by the 64–94 years-old age range, while males start lower at about 1.75 and decline to around 1.40 (Figure 4). The plot of gender*education illustrates that the influence of education is evident, with individuals holding tertiary education showing the lowest mean levels for both conditions, around 1.40 for females and 1.50 for males. In contrast, those with primary education exhibit higher mean levels, peaking at about 1.80 in the 40–50 years-old age group.

## Discussion

### Socio-demographic characteristics of participants

Our study revealed a predominantly female (70.8%), urban (89%) and older adult population (mean age 60.3 years) with limited education (41.3% primary level only) and low socioeconomic status (59.5% earning < 50 000 FCFA/month). This demographic profile aligns with patterns observed in other Cameroonian chronic disease studies.^23^ The female predominance may reflect several factors: greater healthcare-seeking behaviour among women in this context,^25^ higher prevalence of obesity and metabolic syndrome in Cameroonian women^26^ and longer life expectancy for females in the region.^27^

The urban concentration (89%) likely reflects healthcare access disparities, as rural populations face transportation barriers to tertiary centres, limited speciality care availability and greater reliance on traditional medicine.^28,29,30^ The educational profile (only 18.5% with tertiary education) suggests health literacy challenges in medication adherence and self-care, as demonstrated in similar populations.^31^

### Disease prevalence and clinical characteristics

The 41% comorbidity rate exceeds West African averages (32%),^32^ potentially reflecting selection bias at referral centres, true epidemiological transition in urban Cameroon and diagnostic ascertainment bias. The prolonged disease duration (≥ 4 years in 52% – 62% of participants) without adequate control (59% uncontrolled hypertension, 77.8% elevated glucose levels) suggests therapeutic inertia in primary care,^33^ medication adherence challenges^25^ and limited access to guideline-recommended therapies.^34^

### Chronic kidney disease knowledge gaps

Poor recognition of diabetes and hypertension as CKD risk factors (35%) contrasts sharply with the 68% awareness in Nigerian cohorts^35^ and 72% in South African studies.^36^ The persistence of dangerous misconceptions (90% believed urine colour indicated kidney health) despite educational campaigns suggests current approaches fail to account for poor health literacy,^37^ cultural health beliefs^38^ and cognitive biases in risk perception.^39,40^

### Socio-demographic associations with knowledge

The facility-based disparities (Fako Heart Centre: 1.5% vs. Regional Hospital: 16.8%) may reflect variable staff training quality,^41^ differences in patient education materials, or time constraints during consultations. The education gradient (tertiary-educated participants had seven-fold better knowledge) supports calls for pictogram-based education tools, community health worker interventions and mobile health solutions.^28,37,42^

### Self-care practice paradoxes

The salt avoidance paradox (97.3% claimed to avoid salt vs. 92.5% still adding it) could reflect social desirability bias in reporting, unrecognised salt in processed foods or culturally ingrained food preparation practices, as previously observed.^38^ Critically low renal monitoring rates (15.2%) reflect systemic barriers, including equipment shortages,^2^ the absence of national CKD screening policies^1^ and financial constraints.^28^

### Self-care management practices among study participants

The results also revealed significant gaps in self-care practices among high-risk patients in the Buea Health District. While dietary practices showed relatively good adherence (97.3% reported avoiding high-salt foods and 88.5% consumed vegetables regularly), concerning deficiencies were noted in other critical areas. The finding that only 56.5% engaged in regular exercise, with 41.8% never exercising, is particularly alarming given the well-established benefits of physical activity for diabetes and hypertension management.^43^ This prevalence of physical inactivity exceeds rates reported in similar African populations,^44^ suggesting cultural or environmental barriers specific to this region.

The paradox between reported salt avoidance (97.3%) and actual salt addition (92.5%) likely reflects several factors, which may include underestimation of salt content in processed foods, cultural food preparation practices and social desirability bias in self-reported data.^38,45,46^

### Association between self-care practices and clinical factors

The strong association between home glucose monitoring and better outcomes (χ^2^ = 8.64, *p* = 0.003) supports findings from the Steno-2 study that intensive monitoring improves prognosis.^47^ However, the low overall monitoring rates (29.8% for blood pressure, 15.2% for renal function) highlight systemic barriers, including the cost of monitoring equipment, limited health literacy and healthcare provider inertia.^28,31,48^

The protective effect of regular exercise against hypertension (AOR: 0.07, *p* = 0.015) aligns with global evidence,^27^ yet the lack of association with diabetes outcomes suggests that exercise intensity or duration may be insufficient for glycaemic control in this population.

### Clinical and socio-demographic risk factors for chronic kidney disease

Hypertension’s strong independent association with CKD (AOR: 4.28) mirrors findings from the Chronic Renal Insufficiency Cohort (CRIC) study.^49^ The non-significant diabetes-CKD association (AOR: 0.32) contrasts with global data,^1^ possibly because of shorter diabetes duration in our cohort, survival bias (early mortality in poorly controlled diabetics) and inadequate diagnostic sensitivity for diabetic nephropathy.

The age interaction findings (protective effect in the 73–83 years-old age group for diabetes, AOR: –0.17, *p* = 0.035) may reflect selective survival of healthier elderly individuals, cohort effects in disease management and diagnostic detection bias.^36^

### Interaction effects and educational impacts

The consistent protective effect of tertiary education across all interaction plots supports the fundamental cause theory of health disparities.^50^ Gender differences in disease patterns (higher baseline levels but steeper declines in females) may reflect biological risk factors,^51^ healthcare utilisation patterns^25^ and social determinants of health.^52^

### Policy implications

Community-based group exercise programmes can improve chronic disease management. Subsidising home blood pressure and glucose monitoring devices enhances patient self-care. Clear salt labelling on packaged foods supports healthier dietary choices. Visual educational tools can improve health literacy among low-literacy populations. Training nurses in point-of-care CKD screening strengthens early detection capacity. These policies collectively support a proactive, equitable approach to preventing and managing hypertension, diabetes and CKD, particularly in resource-limited settings.

## Conclusion

This study highlights substantial gaps in CKD awareness and self-care practices among high-risk populations in Cameroon. Hypertension emerged as a key modifiable risk factor, while poor adherence to monitoring and lifestyle modifications exacerbates disease progression. Addressing these challenges requires a multi-faceted approach, integrating community-based education, affordable diagnostic tools and strengthened healthcare systems. Policymakers should prioritise preventive strategies, including salt reduction campaigns and subsidised screening programmes, to mitigate CKD’s growing burden. Future research should explore culturally tailored interventions to improve long-term outcomes in resource-limited settings.
